# Adaptive evolution of centromere proteins in plants and animals

**DOI:** 10.1186/jbiol11

**Published:** 2004-08-31

**Authors:** Paul B Talbert, Terri D Bryson, Steven Henikoff

**Affiliations:** 1 Howard Hughes Medical Institute, Fred Hutchinson Cancer Research Center, 1100 Fairview Avenue N, Seattle, WA 98109-1024, USA

## Abstract

**Background:**

Centromeres represent the last frontiers of plant and animal genomics. Although they perform a conserved function in chromosome segregation, centromeres are typically composed of repetitive satellite sequences that are rapidly evolving. The nucleosomes of centromeres are characterized by a special H3-like histone (CenH3), which evolves rapidly and adaptively in *Drosophila *and *Arabidopsis*. Most plant, animal and fungal centromeres also bind a large protein, centromere protein C (CENP-C), that is characterized by a single 24 amino-acid motif (CENPC motif).

**Results:**

Whereas we find no evidence that mammalian CenH3 (CENP-A) has been evolving adaptively, mammalian CENP-C proteins contain adaptively evolving regions that overlap with regions of DNA-binding activity. In plants we find that CENP-C proteins have complex duplicated regions, with conserved amino and carboxyl termini that are dissimilar in sequence to their counterparts in animals and fungi. Comparisons of *Cenpc *genes from *Arabidopsis *species and from grasses revealed multiple regions that are under positive selection, including duplicated exons in some grasses. In contrast to plants and animals, yeast CENP-C (Mif2p) is under negative selection.

**Conclusions:**

CENP-Cs in all plant and animal lineages examined have regions that are rapidly and adaptively evolving. To explain these remarkable evolutionary features for a single-copy gene that is needed at every mitosis, we propose that CENP-Cs, like some CenH3s, suppress meiotic drive of centromeres during female meiosis. This process can account for the rapid evolution and the complexity of centromeric DNA in plants and animals as compared to fungi.

## Background

Centromeres are the chromosomal loci where kinetochores assemble to serve as attachment sites for the spindle microtubules that direct chromosome segregation during mitosis and meiosis. Despite this essential conserved function in all eukaryotes, centromere structure is highly variable, ranging from the simple short centromeres of budding yeast, which have a consensus sequence of approximately 125 base pairs (bp) on each chromosome, to holokinetic centromeres that span the entire length of a chromosome [1]. In plants and animals, centromeres are large and complex, typically comprising megabase-sized arrays of tandemly repeated satellite sequences that are rapidly evolving [2] and may differ significantly between closely related species [3-5]. The failure of conventional cloning and sequencing assembly tools to adequately characterize rapidly evolving satellite sequences at centromeres has made them the last regions of most eukaryotic genomes to be well understood [1].

Although there is no discernable conservation of centromeric DNA sequences in disparate eukaryotes, considerable progress has been made in identifying common proteins that form the kinetochore [6]. A universal protein component of centromeric chromatin found in all eukaryotes that have been examined is a centromere-specific variant of histone H3 (CenH3), which replaces canonical H3 in centromeric nucleosomes [7,8]. CenH3s are essential kinetochore components yet, like centromeric DNA, they are rapidly evolving [1]. In both *Drosophila *[9] and *Arabidopsis *[10], this rapid evolution of CenH3s is associated with positive selection (adaptive evolution), and involves regions of CenH3 that are predicted to contact the centromeric DNA [9,11,12].

The finding of positive selection in a protein that is required at every cell division is remarkable. Ancient proteins with conserved function are expected to be under negative selection because they typically have achieved an optimal sequence, so new mutations tend to produce deleterious variants that are quickly eliminated from populations. The canonical histones are extreme examples of this type of protein. In contrast, recurrent positive selection generally occurs as a consequence of genetic conflict, for example in the 'arms race' between pathogen surface antigens and the immune-cell proteins that recognize them. In this case, a mutation in a surface antigen that allows the pathogen to escape detection and proliferate will trigger selection for a new immune receptor to fight the mutated pathogen, which can then mutate again, and so on. The evidence for positive selection of CenH3 proteins specifically in the regions that contact DNA thus suggests a conflict between centromeric DNA and a histone component of the nucleosome that packages it. Is it commonplace for eukaryotes to have such a conflict at their centromeres? Is the conflict unique to centromere-specific histones, or are other proteins that bind centromeres also involved in this conflict? Is conflict responsible for centromere complexity? To answer these questions, we investigated the evolution of a second common DNA-binding kinetochore protein.

Of the handful of essential kinetochore proteins that are widely distributed among eukaryotes, only one class other than CenH3 has been shown to bind centromeric DNA: centromere protein C (CENP-C), a conserved component of the inner kinetochore in vertebrates [13-16]. Human CENP-C binds DNA non-specifically *in vitro *[17-19] and binds centromeric alpha satellite DNA *in vivo *[20,21]. Vertebrate CENP-C and the yeast centromere protein Mif2p [22,23] share a 24 amino-acid motif (CENPC motif) that has also been found in kinetochore proteins in nematodes [24] and plants [25]. As expected for kinetochore proteins, disruption or inactivation of genes encoding proteins containing a CENPC motif (CENP-Cs) results in the failure of proper chromosome segregation [16,23,24,26-28].

Other than the defining CENPC motif, these proteins are dissimilar in sequence across disparate phyla. Such a small stretch of sequence conservation, accounting for less than 5% of the length of these 549-943 amino-acid proteins, is unexpected considering that CENP-Cs are encoded by essential single-copy genes that are expected to be subject to strong negative selection. We therefore wondered whether the same evolutionary forces responsible for the rapid evolution of CenH3s cause divergence of CENP-Cs outside of the CENPC motif.

Here, we describe coding sequences from several unreported *Cenpc *genes and test whether *Cenpc *genes are in general, like *CenH3 *genes, subject to positive selection. We find evidence for adaptive evolution of CENP-C in plants and animals, but we find negative selection in yeasts. Our results provide support for a meiotic drive model of centromere evolution.

## Results and discussion

### CenH3s evolve under negative selection in some lineages

Previous work has shown that CenH3s are evolving adaptively in *Drosophila *and *Arabidopsis *[9,10], but their mode of evolution in mammals is not known. Selective forces acting on proteins can be measured by comparing the estimated rates of nonsynonymous nucleotide substitution (K_a_) and synonymous substitution (K_s_) between coding sequences from closely related species. These rates are expected to be equal if the coding sequences are evolving neutrally (K_a_/K_s _= 1). Negative selection is indicated by K_a_/K_s _< 1, and positive selection is indicated by K_a_/K_s _> 1.

**Figure 1:**
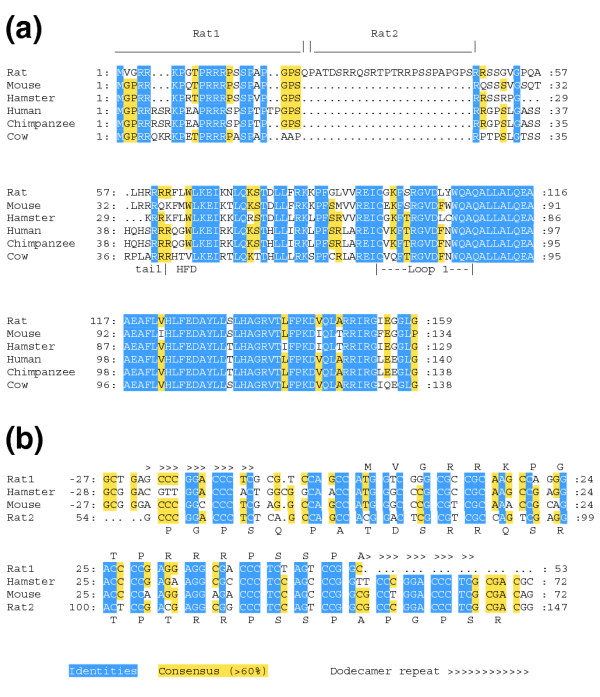
The rat CENP-A protein. **(a) **Alignment of predicted CENP-A proteins of mammals. Relative to other mammalian CENP-As, rat CENP-A has a 25 amino-acid insertion that arises from a duplication of the amino terminus, shown as over-lined regions. The boundary between the tail and the histone-fold domains (HFD) is indicated below the alignment, along with the position of Loop 1. **(b) **Alignment of duplicated regions of the rat *Cenpa *gene (rat1 and rat2) with *Cenpa *genes of mouse and Chinese hamster. The region that became duplicated in rat extends from upstream of the start codon to codon 22 in mouse and hamster, and is bounded by a conserved dodecamer repeat. The encoded amino acids are shown above (rat1) or below (rat2) the duplicated sequence.

To obtain a pair of closely related mammalian CenH3s, we used the sequence of the mouse (*Mus musculus*) CenH3, CENP-A [29], to query the High Throughput Genomic Sequences portion of the GenBank database [30] with a tblastn search, and identified a rat (*Rattus norvegicus*) genomic clone (AC110465) that contains the predicted rat CENP-A coding sequence. The predicted CENP-A protein is encoded in four exons and is 87% identical in amino-acid sequence to mouse CENP-A, excluding a 25 amino-acid insertion that appears to derive from a duplication of the amino terminus (Figure 1). This gene model is partially supported by an expressed sequence tag (EST; BF561223) that includes the first three exons, but which terminates in the predicted intron 3.

To determine whether *Cenpa *is evolving adaptively in rodents, we compared K_a _and K_s _between mouse and rat using K-estimator [31]. Positive selection in single-copy genes that are essential in every cell is expected to be localized and more difficult to detect than in nonessential genes or members of multigene families because of simultaneous negative selection to maintain their essential functions. In *Drosophila *and *Arabidopsis*, CenH3s are under positive selection in their tails, but also under negative selection in much of their histone-fold domains. We therefore used the sliding-window function of K-estimator to scan through the coding sequences using 99 bp windows every 33 bp in an effort to find regions of positive selection. This analysis detected statistically significant negative selection for all of the windows except one that failed to rule out neutrality, indicating that CENP-A is under negative selection (K_a _= 0.11, K_s _= 0.33; K_a _< K_s _with *p *< 0.001) in both the tail and the histone-fold domains. Similar results were obtained when comparing either sequence with the *Cenpa *gene from Chinese hamster (*Cricetulus griseus*) [32], although the greater divergence (K_s _= 0.45 rat, 0.67 mouse) makes the statistical conclusion near the limit of reliability (K_s _≤ ~0.5) because of the increased likelihood of multiple substitutions. Thus, CENP-A appears to have been under negative selection throughout its length in multiple rodent lineages.

We also compared the human *Cenpa *gene [33] with the *Cenpa *gene from chimpanzee (*Pan troglodytes*). A blastn search of the Genome Sequencing Center's assembly of the chimpanzee genome [34] using human *Cenpa *identified the chimp *Cenpa *gene encoded in four exons in Contig 286.218. We searched the NCBI trace archives [35] to verify the sequence and the existence of appropriate putative intron splice sites. The predicted chimpanzee *Cenpa *gene differs from the human gene by six synonymous nucleotide substitutions and an indel (insertion or deletion) of two codons. This excess of synonymous substitutions indicates negative selection of CENP-A (*p *< 0.01). Overall negative selection of CENP-A appears also to extend to the bovine (CB455530) protein, given the relatively high degree of conservation seen for all regions, including the tail and Loop 1 regions that evolve adaptively in *Drosophila *(Figure 1a).

We also found overall negative selection in CenH3s of grasses. We used the *CENH3 *gene (AF519807) of maize (*Zea mays*) [36] to search ESTs [37] from sugarcane (*Saccharum officinarum*), and identified three that encode full-length *CENH3 *genes (CA119873, CA127217, and CA142604). The CenH3 proteins encoded by these ESTs differ from each other by 2-4 amino acids. Because sugarcane is thought to be octaploid, these variants may represent co-expressed homeologs. The coding regions of ESTs CA119873 and CA127217 differ by four synonymous and four nonsynonymous substitutions (K_s _= 0.03, K_a _= 0.01), suggesting negative selection. Comparison of either of these sequences with maize *CENH3 *by sliding-window analysis found that all windows had K_s _> K_a_, with overall negative selection (K_s _= 0.24, K_a _= 0.13; *p *< 0.01). Thus, in contrast to CenH3s in *Arabidopsis *and *Drosophila*, CenH3s of rodents, primates, and grasses appear not to be evolving adaptively.

The evident lack of positive selection on CenH3 in mammals and grasses raises the possibility that another kinetochore protein is evolving in conflict with centromeric DNA in these organisms, in which centromeric satellite sequences are known to be evolving rapidly [2,38]. We focused on CENP-C, which is found to co-localize with CenH3 to the inner kinetochore in humans [13] and maize [36].

### Mammalian CENP-C is evolving adaptively

**Figure 2:**
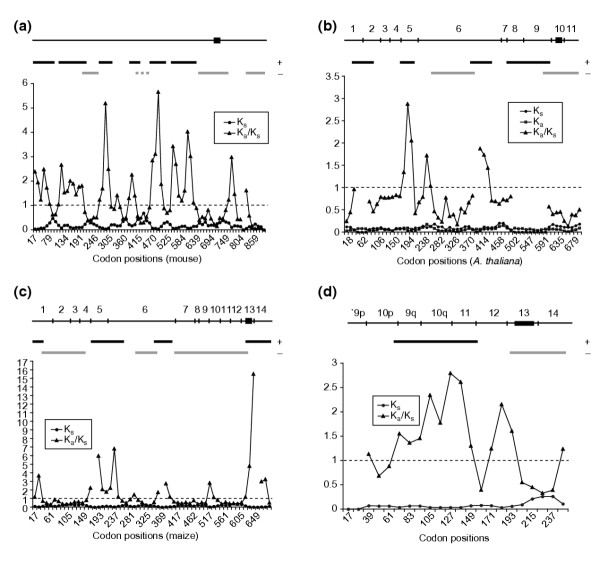
Sliding-window analysis of K_a_/K_s _for selected pairs of *Cenpc *genes. Each point represents the value of K_s_, K_a_, or K_a_/K_s _for a 99 nucleotide (33 codon) window plotted against the codon position of the midpoint of the window. K_a_/K_s _is not defined where K_s _= 0. The aligned coding sequence is represented at the top of each graph, with the CENPC motif represented by a filled rectangle; exons are also indicated for the plant sequences. Regions of statistically significant positive selection (black bars) and negative selection (gray bars) are marked. **(a) **Rat and mouse. The interrupted gray bar indicates that *p *= 0.06 for this region. **(b) ***Arabidopsis thaliana *and *Arabidopsis arenosa*. **(c) **Maize (*CenpcA*) and *Sorghum bicolor*. **(d) **Wheat and barley, exons 9p-14.

To address the possibility that CENP-C is adaptively evolving in mammals, we used the mouse sequence [14] as a query in a tblastn search to identify *Cenpc *ESTs from rat. From these ESTs (see Additional data file 1), we obtained and sequenced a full-length cDNA (see Additional data file 2), and compared its coding sequence with that of the mouse *Cenpc *gene (68% predicted amino-acid identity). We found positive selection over most of the amino-terminal two-thirds of the coding sequence, interrupted by one region of significant negative selection (mouse codons 208-273), one region of nearly significant negative selection (mouse 410-464), and three short regions without significant selection (Figure 2a; Table 1). Most of the carboxy-terminal one-third of the protein, including the CENPC motif and an additional region that is homologous to the budding yeast CENP-C protein Mif2p [22,23], has been under negative selection. We conclude that at least some regions of *Cenpc *genes are evolving adaptively in rodents.

To determine whether any of these regions is also under positive selection in primates, we identified the *Cenpc *gene of chimpanzee by using the human *Cenpc *coding sequence (GenBank accession number M95724) to search the assembled chimpanzee genome and the NCBI trace archives. We found that the chimpanzee genome contains a single copy of the *Cenpc *structural gene (contigs 375.88-375.100), as well as a processed *Cenpc *pseudogene (contigs 76.642-76.643), as has been found in humans [14,18,39]. The predicted chimpanzee *Cenpc *coding sequence differs by 17 nucleotide substitutions from the human cDNA sequence, with K_s _= 0.0054 and K_a _= 0.0063. The > 99% identity of the human and chimp coding sequences provides little opportunity to detect selection, but using sliding-window analysis we found a single region of significant positive selection (human codons 278-585) that overlaps the central regions of positive selection found in the more divergent rat-mouse comparison, indicating that the central portion of CENP-C is under positive selection in both rodents and primates.

To confirm these results, we applied the codeml program of PAML [40] to a multiple sequence alignment of mammalian CENP-Cs. PAML calculates the likelihood of models for neutral and adaptive evolution based on a tree and estimates K_a_/K_s _ratios. We compared the null model with two fixed site classes (K_a_/K_s _= 0 or 1) to a 'data-driven' model in which two classes of sites were estimated from the data. The data-driven model was found to be significantly more probable than the null model (χ^2 ^= 8.7; *p *= 0.01) with K_a_/K_s _= 0.20 for 57% of the 685 sites in the multiple alignment and K_a_/K_s _= 1.64 for 43% of the sites (data not shown). Similar results were obtained using either a DNA- or a protein-based tree, or testing more complex models. When the same tests were applied to the core region of 11 aligned Brassicaceae (mustard family) CenH3s, only 17% of residues were estimated to be in the positive selection class (K_a_/K_s _= 2.54) ([11] and data not shown), which indicates that positive selection on mammalian CENP-C has occurred more extensively than on CenH3s.

Amino-acid sites of positive selection in mammalian CENP-Cs were identified as those with significant posterior probabilities. These were found to be scattered throughout the multiply aligned region with 5 of the 18 highly significant sites prominently clustered within 25 residues (human codons 424-448) in a region of positive selection identified by K-estimator analysis. Therefore, pairwise K-estimator and multiple PAML analyses yield similar results and reveal that large regions of mammalian CENP-Cs have been adaptively evolving.

### Adaptively evolving regions overlap DNA-binding and centromere-targeting regions

**Figure 3:**
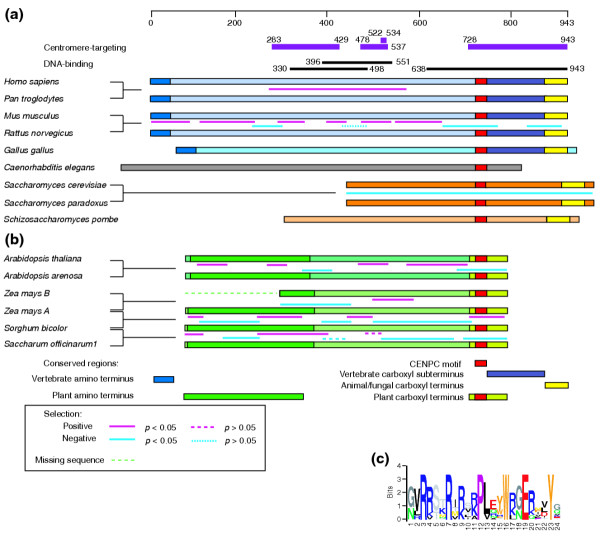
Comparisons of CENP-C proteins in animals, yeast and plants. The CENPC motif and conserved regions found at the termini of CENP-C proteins are indicated. For pairwise comparisons of protein-coding sequences, regions of positive and negative selection between the species compared are shown. **(a) **Alignment of animal and fungal CENP-Cs. Mammalian CENP-Cs align throughout their lengths, as do the two *Saccharomyces *Mif2p proteins, but others align only at conserved regions. Portions of the human CENP-C protein implicated in centromere-targeting (purple bars) and DNA-binding (black bars) are shown at the top. The scale bar at the top marks the length of human CENP-C in amino acids. **(b) **Alignment of plant CENP-Cs. Within angiosperm families, proteins align throughout their lengths. Between families, weak conservation is found at the amino terminus and strong conservation at the carboxyl terminus. **(c) **Logos representation of an alignment of the CENPC motif from human; mouse; cow; chicken; *Caenorhabditis elegans*; budding yeast; *Schizosaccharomyces pombe*; *Physcomitrella patens*; maize *CenpcA*; rice; *A. thaliana*; black cottonwood, soybean, and tomato.

The regions of positive selection in rodent and primate CENP-Cs overlap some protein landmarks identified in functional analyses of human CENP-C. The binding activity of human CENP-C to DNA *in vitro *has been mapped by two groups of investigators. Sugimoto and colleagues [17,18] found that the region including amino acids 396-498 bound DNA and was stabilized by including flanking amino acids on one or both sides (330-498 or 396-581; Figure 3a), suggesting that at least two regions in the central portion of the protein contribute to DNA binding. Yang and colleagues [19] identified two non-overlapping DNA-binding regions: amino acids 23-440 and 459-943. They found a weak DNA-binding activity at the carboxyl terminus in region 638-943, which includes the CENPC motif (737-759) and the conserved Mif2p-homologous region (890-941). This suggests that region 459-943 itself contains at least two DNA-binding regions, a weak one at region 638-943, and a stronger one that may correspond to region 396-581 described by Sugimoto and colleagues. Both the central region and the carboxyl terminus have been shown to bind DNA *in vivo *[21]. Comparison of the regions of positive selection found in rodents and primates with these DNA-binding regions reveals extensive overlap with the central DNA-binding regions (Figure 3a), including the cluster of highly significant sites between codons 424 and 448 identified by PAML analysis. This is consistent with previous evidence that adaptive evolution of CenH3s occurs in regions that have been implicated in DNA binding [9,11]. No positive selection was observed for the poorly mapped carboxy-terminal DNA-binding domain in our sliding-window analysis, suggesting either that this DNA-binding domain is not evolving adaptively or that strong negative selection on the CENPC motif can obscure detection by our sliding-window analysis of positive selection on nearby amino acids that contact centromeric DNA. In the DNA-binding Loop 1 region of *Arabidopsis *CenH3, adaptively evolving codons are found in close proximity to codons under strong negative selection [11].

In human CENP-C, three regions have been reported to confer centromere targeting. One targeting signal was recently reported in region 283-429 [41]. A second targeting region was mapped by mutation to region 522-534, with arginine 522 crucial for localization [42]. Targeting by the conserved carboxyl terminus (728-943) occurs for species as distant as *Xenopus *[21,41-43]. A segment that includes both the first and second targeting regions (1-584) failed to confer targeteting to centromeres in hamster BHK cells, however [43]. We find that these two targeting regions are within the region of positive selection in primates and overlap with three of the regions of positive selection in rodents. A correspondence between centromere targeting and adaptive evolution has been noted for *Drosophila *CenH3, where the adaptively evolving Loop 1 region has been shown to be necessary and sufficient for targeting when swapped between native and heterologous orthologs [44]. Therefore, the lack of centromeric targeting of a human CENP-C fragment containing the first and second targeting regions in the heterologous hamster system might be attributed to adaptive evolution of DNA-binding specificity in these regions.

Targeting of native CENP-C proteins depends on other centromere proteins that vary according to species [45], but the dependence of CENP-Cs on CenH3s for targeting appears to be universal [24,46-49]. This dependence suggests that CENP-C proteins contain a conserved CenH3-interacting region, for which the CENPC motif is the only obvious candidate. The first half of the CENPC motif is rich in arginines, whereas the second half has mixed chemical properties including three aromatic residues (Figure 3c). In the non-specific binding of nucleosome cores to DNA, 14 DNA contacts are made by arginines binding to the minor groove [50]. This suggests that the weak DNA binding of the carboxyl terminus of CENP-C may be mediated by the arginines of the CENPC motif, with the remainder of the motif contacting a conserved structural feature of centromeric nucleosomes.

Not all regions of CENP-C that display positive selection correspond to regions that bind DNA *in vitro *or that are sufficient for targeting centromeres. For example, the region comprising the most amino-terminal 200 or so amino acids of rodent CENP-C has been evolving adaptively, but the orthologous region in human CENP-C fails to bind DNA in a southwestern assay [17,19] or to localize to centromeres of human embryonic kidney cells [21]. This suggests that the amino-terminal region of CENP-C plays a supporting role in packaging centromeric chromatin. A parallel situation appears to hold for the adaptively evolving amino-terminal tail of *Drosophila *CenH3, which was found to be neither necessary nor sufficient for targeting *in vivo *to homologous centromeres. In this case, Loop 1 was identified as the targeting domain, and the amino-terminal tail was hypothesized to help stabilize higher-order chromatin structure by binding to linker DNA, similar to the known binding activity of canonical histone tails [44]. If CENP-C in mammals is subject to the same evolutionary forces that shape the adaptive evolution of the CenH3 tail in *Drosophila*, then CENP-C might be playing a comparable role in the stabilization of higher-order centromeric chromatin.

Positive selection in the central DNA-binding and centromere-targeting region of CENP-C offers an explanation for the lack of conservation of this region between chicken and mammals [51]: as positive selection acts on the amino acids that contact rapidly evolving centromeric satellites and that serve to target the protein to a specific but ever-changing substrate, it may eventually erase all recognizable homology in these protein regions.

### *Cenpc *gene structure and conservation in plants

Our finding that adaptive evolution is occurring in animal CENP-Cs encouraged a similar survey of plant CENP-Cs, because centromeres from both animals and seed plants comprise rapidly evolving satellite sequences. At the time we began this study, *Cenpc *genes in plants had been characterized only in maize (*Z. mays*), so we needed first to identify *Cenpc *homologs from other plants to ascertain whether or not the gene is evolving adaptively.

**Figure 4:**
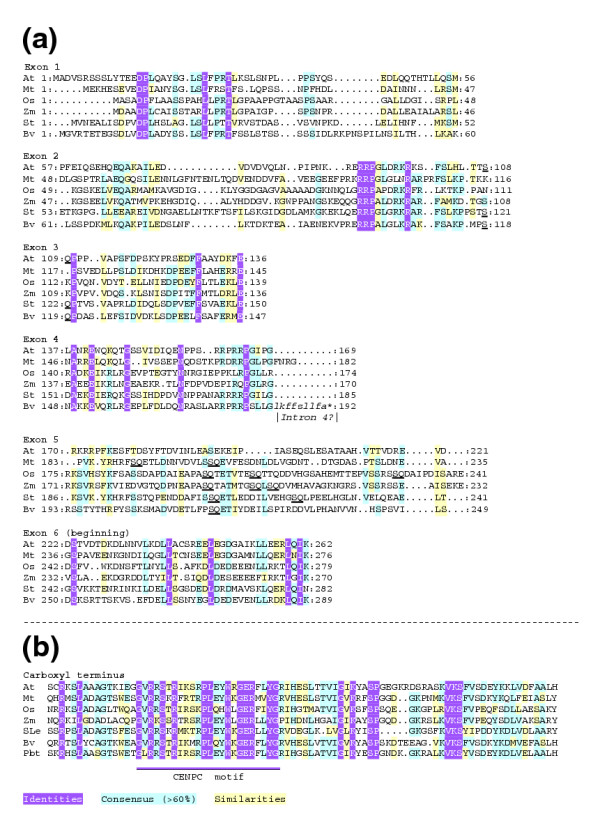
Alignment of conserved regions of angiosperm CENP-C predicted proteins. **(a) **Short regions of conservation are encoded in the first six exons of *Cenpc *genes from five families. The dipeptide SQ (underlined) is relatively frequent in exon 5. **(b) **Multiple alignment reveals strong conservation in the carboxyl termini of encoded proteins from six families. The CENPC motif is indicated. At, *A. thaliana*; Mt, barrel medic; Os, rice; Zm, maize CENP-CA; St, potato; SLe, tomato; Bv, beet; Pbt, black cottonwood.

**Figure 5:**
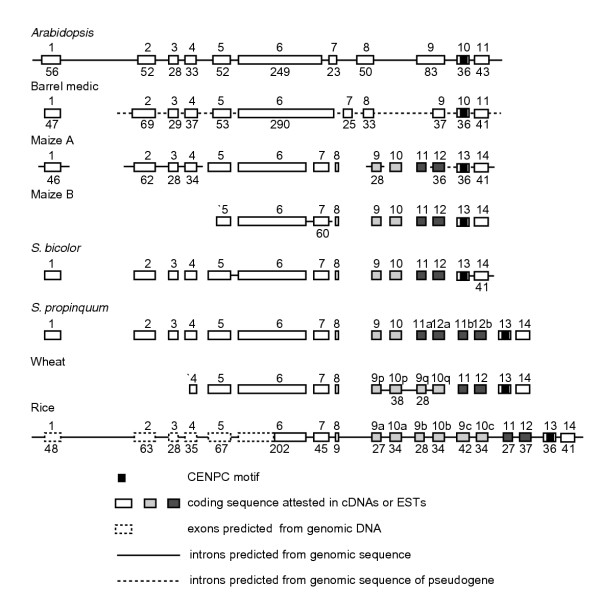
Gene models of selected plant *Cenpc *genes. Exon/intron structure is conserved across families from exon 1 through the beginning of exon 6, and for the final two exons and introns. Exon sizes are given to the nearest codon where genomic sequence is available to confirm predicted exons. Duplicated exons are indicated by gray shading.

Three *Cenpc *homologs have been described in maize: *CenpcA, CenpcB, *and *CenpcC *[25]. Immunological localization of CENP-CA to maize centromeres indicates that it is probably functional, so plant relatives of maize CENP-CA should also represent CENP-Cs. We used the CENP-CA protein sequence (AAD39434) as a query in a tblastn search of GenBank, and identified a single *Cenpc *homolog (AC013453, At1g15660) in the genome of *Arabidopsis thaliana *by sequence similarity at both protein termini (Figure 4). Isolation and sequencing of a full-length *Cenpc *cDNA (Additional data file 2) revealed that the 705 amino-acid CENP-C protein of *Arabidopsis *is encoded in 11 exons, with the CENPC motif encoded in exon 10 (Figure 5). Recently, *Arabidopsis *CENP-C has been found to localize to *Arabidopsis *centromeres [52].

We searched the GenBank EST database, querying with the predicted protein sequences of maize CENP-CA and *Arabidopsis *CENP-C. We identified ESTs from putative plant *Cenpc *genes in 20 angiosperm species representing eight families and in the moss *Physcomitrella patens *(see Additional data file 1). We obtained the cDNA clones corresponding to 16 of these ESTs and sequenced them completely (see Additional data file 2). An alignment of the carboxyl termini encoded by cDNAs representing six angiosperm families revealed that the final 80 or so amino acids of CENP-C, including the CENPC motif, are highly conserved in plants (Figure 4b). For comparison, the carboxyl termini of vertebrate CENP-C proteins have approximately 180 amino acids following the CENPC motif (Figure 3a), including a block of 52 amino acids that is conserved in yeast Mif2p [22,23], but not in nematodes [24]. The carboxyl termini of plant CENP-Cs do not show significant similarity to animal and fungal CENP-Cs except for the CENPC motif.

As an aid in identifying other conserved regions of angiosperm CENP-Cs, we developed gene models for full-length *Cenpc *cDNAs by aligning them with available genomic sequences (Additional data file 1). A full-length cDNA from barrel medic (*Medicago truncatula*) encodes a protein of 697 amino acids, which corresponds to a gene model of eleven exons when aligned to a genomic pseudogene (Figure 5). We also predicted gene models for *Cenpc *genes in the grasses using cDNAs and genomic sequences from rice (*Oryza sativa*), maize, and sorghum (*Sorghum bicolor*) (Figure 5). The maize gene model of 14 exons suggests an explanation for the anomalous maize cDNA '*CenpcC*' (AF129859) [25], which differs from all other plant *Cenpc*s in encoding an unrelated carboxyl terminus. *CenpcC *is 99.9% identical to maize *CenpcA *until it diverges downstream of the CENPC motif at the point corresponding to the end of exon 13 in our gene model. On the basis of an overlap with maize and *Sorghum *genomic sequence that spans the intron between exons 13 and 14, we conclude that the divergent 3' end of *CenpcC *derives from the unspliced intron 13 of *CenpcA*, and that all angiosperm CENP-Cs share a highly conserved carboxyl terminus.

Comparing the gene models of *Arabidopsis*, barrel medic, maize, *Sorghum*, and rice, the limited conservation of the encoded amino-acid sequences and approximate correspondence of exon sizes suggest that the exons in the amino-terminal half and the final two exons of plant CENP-C are conserved (Figures 3,5). The middle region does not show conservation of intron position or encoded peptide sequence, indicating rapid evolution within angiosperms. We assumed conservation of the first five intron positions in the 5' half of the coding sequence to generate an amino-terminal alignment that represents five families, including the protein encoded by a beet (*Beta vulgaris*) cDNA that appears to contain an unspliced intron. Our alignment reveals short regions of conservation throughout the amino terminus, as well as a high relative incidence of the dipeptide SQ in the poorly conserved exon 5 (Figure 4).

Despite these short regions of conservation within angiosperms, no sequence similarity between plant and animal CENP-Cs could be detected outside of the CENPC motif. Nevertheless, plant and animal CENP-Cs appear to share an overall architecture (Figure 3). Both angiosperm and vertebrate CENP-Cs [16] have regions of conservation at the amino and carboxyl termini, with little or no conservation in the middle region of the protein. Remarkably, plant and animal CENP-Cs also share the same modular exon organization for the CENPC motif, which lies within a 105-108 bp exon (encoding 35-36 amino acids) that is spliced in the same frame in both plants and animals (see Additional data file 3). Considering the similar overall lengths of plant and animal CENP-Cs, the arrangement of conserved regions, and the common location of the CENPC module, it appears that corresponding regions of the protein are evolving similarly and may serve similar functions.

### Recurrent exon duplications in the grasses

Multiple alignment of plant *Cenpc*s revealed that one region of the gene is subject to duplication, but only in grasses. One part of the poorly conserved middle region of the gene has been repeatedly duplicated and deleted, thus encoding proteins of different sizes. In rice, an ancestral pair of exons, corresponding to exons 9 and 10 in maize *CenpcA*, has been triplicated in tandem (Figure 5). To facilitate comparison with maize and other grasses, we designated the rice exons as 9a-10a, 9b-10b, and 9c-10c. Exon 9c has an additional internal tandem duplication of its first 14 codons. Consensus sequences derived from overlapping truncated ESTs (Additional data file 1) and cDNAs (Additional data file 2) from the closely related species wheat (*Triticum aestivum*) and barley (*Hordeum vulgare*) indicate that there are two tandem copies of exons 9 and 10 in these species (designated 9p-10p and 9q-10q in Figure 5). We confirmed the sequence of these exons by designing primers and amplifying the corresponding regions from wheat and barley genomic DNAs. Single copies of exons 9 and 10 were found in full-length cDNAs from sugarcane, *Sorghum bicolor *and *Sorghum propinquum *(Table 2; Figure 5).

Exon duplications were also found for *Sorghum *species but, surprisingly, these involved a different pair of exons, 11 and 12. One full-length cDNA from *S. bicolor *has only a single copy of exons 11 and 12, whereas a truncated pseudogene from *S. bicolor *and a full-length cDNA from *S. propinquum *are duplicated for exons 11 and 12 (designated 11a-12a and 11b-12b). The *S. bicolor *pseudogene has a deletion that joins sequences just upstream of the initiation codon in exon 1 to sequences upstream of exon 2. Despite the presence of tandemly duplicated exons, the *S. bicolor *truncated pseudogene is more closely related to the full-length *S. bicolor *gene than it is to the *S. propinquum *gene. Exons 11 and 12 in the *S. bicolor *full-length gene are identical to 11b-12b in the pseudogene, but have 7 differences from 11a-12a. This suggests that the duplication of exons 11 and 12 preceded the divergence of *S. propinquum *and *S. bicolor*, and that the full-length *S. bicolor *gene may have been derived by loss of exons 11a-12a from a full-length ancestral gene similar to the truncated pseudogene.

**Figure 6:**
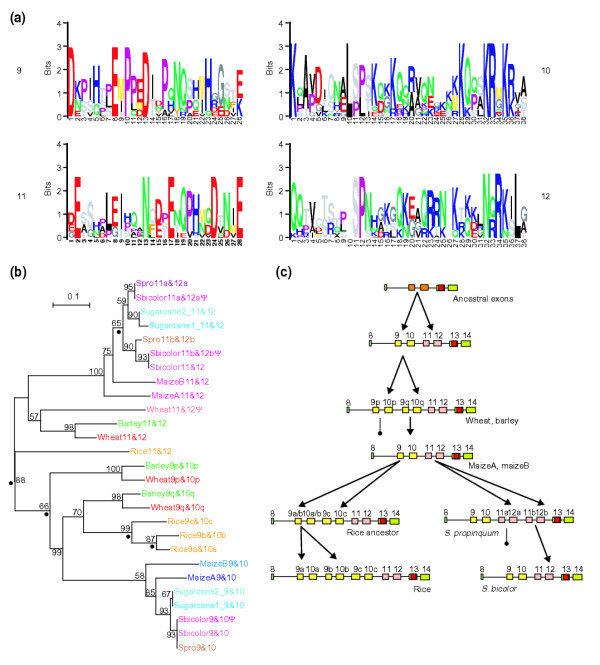
CENP-C exon repeats in the grasses. **(a) **Alignments of copies of the duplicated exons 9, 10, 11, and 12 from the grass species in this study, excluding pseudogenes, are shown in Logos format. **(b) **A neighbor-joining phylogram (with gaps excluded) of the exon pairs 9-10 and 11-12 in grass species. A parsimony tree gave essentially the same topology. Dots indicate the locations of inferred duplication events in the tree. Presumed pseudogenes are marked with ψ. **(c) **Schematic representation of exon duplication events leading to various *Cenpc *gene structures, and examples of grass species with these structures. Pairs of arrows indicate duplication events; lines terminating in a filled circle indicate loss of an exon pair in derivatives.

We wondered why two different pairs of exons, 9-10 and 11-12, were each independently subject to duplication in the grasses. When we examined multiple alignments of the peptide sequences encoded by both exon pairs in Logos format, it became apparent that they resembled each other in length and composition (Figure 6a). Exons 9 and 11 both encode peptides of 25-28 residues that are rich in acidic amino acids, whereas exons 10 and 12 encode peptides of 30-38 residues that are rich in basic amino acids. We compared alignments of exons 9 and 11 and alignments of exons 10 and 12 using the Local Alignment of Multiple Alignments (LAMA) program, and found that these exon pairs appear to be homologous (*E *< 0.0001 for both comparisons). We conclude that exon pairs 9-10 and 11-12 derive from a more ancient duplication event.

To trace the likely ancestry of these duplication events, we used an alignment of the exons from multiple species to construct phylogenetic trees of duplicates of exons 9-10 and 11-12 (Figure 6b). This phylogeny suggests that there have been numerous duplication events in the history of the grasses (Figure 6c and data not shown): first, a duplication generating exons 9-10 and 11-12 in an ancestor of the grasses; second, a duplication generating exons 9p-10p and 9q-10q; third, a duplication generating exons 11a-12a and 11b-12b in the *Sorghum *lineage; fourth, two duplications generating rice exons 9a-10a, 9b-10b, and 9c-10c all within the rice 9q-10q lineage; and fifth, a partial duplication in rice exon 9c.

There also appear to have been at least three losses of duplications: one of exons 11a-12a in the lineage leading to the full-length *S. bicolor *gene, one of exons 11b-12b in the sugarcane genes, and one of the hypothetical rice 9p-10p. Alternatively, it is possible that the latter loss and one of the rice-specific duplications resulted from gene conversion of rice 9p-10p by a derivative of rice 9q-10q. Regardless of the exact number of duplication and deletion events, it is clear that the exon pair ancestral to grass exons 9-10 and 11-12 has been subjected to repeated episodes of duplication and deletion.

### Plant CENP-Cs are adaptively evolving

The delineation of gene models for plant *Cenpc*s allowed us to analyze them for evidence of adaptive evolution. First, we compared *Cenpc*s from *Arabidopsis *species in which we had previously found adaptively evolving CenH3s. Using the *A. thaliana *genomic sequence to design primers, we amplified, cloned, and sequenced a *Cenpc *cDNA from *A. arenosa *(Additional data file 2). Comparing this sequence with that of *A. thaliana, *the predicted proteins differ by 87 amino-acid subtitutions out of 703 alignable residues, plus five indels of 1-3 amino acids.

We applied the sliding window option of K-estimator to the aligned coding sequences of *A. thaliana *and *A. arenosa Cenpc*. At three regions, K_a _exceeded its 99% confidence interval for the null hypothesis, indicating that these regions are under positive selection (Figures 2b,3). These regions correspond approximately to exon 5 (codons 178-221 in the *A. thaliana *sequence), the 3' half of exon 6 (codons 376-441), and exons 8 and 9 (codons 486-618). In addition, a region encompassing most of exons 1 and 2 (codons 24-89) was found to be under positive selection with *p *< 0.03. We also determined that the 5' half of exon 6 (codons 255-386) and the conserved exons 10 and 11 (codons 595-703) are under negative selection with *p *< 0.01.

Curiously, an indel at the beginning of exon 9, where the *A. arenosa *cDNA has a CAG (glutamine) codon that is absent in the *A. thaliana *cDNA, appears to be caused by the species-specific use of alternative acceptor splice sites, because the genomic sequence (data not shown) at this intron-exon boundary is identical in both species (...cag cag ^GAG GGT... or ...cag ^CAG GAG GGT...). The presence of species-specific alternative splicing of the same codon in an adaptively evolving region suggests that splicing variation can contribute to adaptive variability.

To examine whether positive selection in *Cenpc *is unique to *Arabidopsis *or occurs more generally in plants, we compared *Cenpc *genes from the two *Sorghum *species. We removed the duplicate exons 11a and 12a from the *S. propinquum *coding sequence in order to compare the sequence with the full-length gene from *S. bicolor *. For this comparison, K_a _= 0.014 and K_s _= 0.003. K_a _exceeds the 99% confidence interval of the null hypothesis, and neutral evolution can be rejected in favor of positive selection. The limited divergence between these two sequences did not allow statistically significant conclusions to be drawn about positive selection in particular regions of the gene.

To address which regions are under positive selection, we compared the *S. bicolor *sequence with the maize *CenpcA *coding sequence (75% amino-acid identity). Between maize *CenpcA *and *S. bicolor, *K_a _= 0.12, K_s _= 0.14, and there are seven indels of 1-11 codons. We identified positive selection for a single window in exon 1, for a region including all of exon 5, for a region in the second half of exon 6, and for a region from the end of exon 12 through most of exon 14 (Table 2 and Figure 2c). Negative selection was found for a region from exons 1-4, a region in the middle one-third of exon 6, and a region from the end of exon 6 through exon 12 (Table 2 and Figure 2c). The regions of positive selection seen in exons 1 and 5 clearly overlap the corresponding regions in *Arabidopsis *(Figure 3b). Although the region of positive selection seen in exon 6 of the grasses cannot be aligned with that in exon 6 of *Arabidopsis *because of sequence divergence, they occur in the same general area of the protein.

The region of positive selection in exons 12-14 was somewhat surprising given the strong conservation around the CENPC motif, and we wondered if this selection was specific to the maize or *Sorghum *lineage. To test this possibility, we compared maize *CenpcA *and *S. bicolor Cenpc *with a *Cenpc *gene from sugarcane. Of three sugarcane cDNAs that we obtained (Additional data file 2), two had identical coding sequences (*Cenpc1*), and the third (*Cenpc2*) differed by 13 nucleotide substitutions, suggesting that *Cenpc1 *and *Cenpc2 *may be homeologous genes in the polyploid sugarcane genome. We compared *Cenpc1 *to maize *CenpcA *and *S. bicolor Cenpc*. Regions of positive or negative selection identified in the maize/*Sorghum *comparison were generally found to coincide with regions under selection in the corresponding direction in maize/sugarcane and *Sorghum*/sugarcane comparisons, although in a few cases the selection was not found at the *p *< 0.05 level of significance in all comparisons (Table 2). We conclude that these regions are subject to recurrent adaptive evolution.

In two cases, a region was found to be under significant selection in opposite directions in different comparisons. First, a region in exon 6 (maize codons 232-286) that was not under significant selection in the maize/*S. bicolor *comparison was under negative selection in the maize/sugarcane comparison, but under positive selection in the *S. bicolor*/sugarcane comparison; this suggests that positive selection in *S. bicolor *and negative selection in maize combined to give a non-significant result in the maize/*S. bicolor *comparison. Second, the region of positive selection in exons 12-14 identified from the maize/*Sorghum *comparison was under positive selection in the maize/sugarcane comparison, but under negative selection in the *Sorghum*/sugarcane comparison, indicating that the positive selection in this region is unique to the maize *CenpcA *lineage (Table 2, Figure 3b). Therefore, in some regions of CENP-C adaptive evolution appears to be episodic, as has been seen previously for primate lysozymes [53].

Phylogeny-based PAML analysis confirms that plant CENP-Cs are adaptively evolving and in an episodic fashion, consistent with inferences based on pairwise K-estimator analysis. As was found for mammalian CENP-C, the data-driven model for grass CENP-C was found to be significantly more probable than the null model (χ^2 ^= 12.0; *p *= 0.003) with K_a_/K_s _= 0.00 for 51% of the 686 sites in the multiple alignment and K_a_/K_s _= 2.00 for 49% of the sites (data not shown). Using the PAML 'free-ratio' option that measures K_a_/K_s _differences between branches in a tree [54], we found that CENP-C is adaptively evolving (χ^2^= 10.0; *p *= 0.007 for the data-driven over the null model) along both *Sorghum *lineages (K_a_/K_s _= 2.6) and along the sugarcane *Cenpc2 *lineage (K_a_/K_s _= 1.3) but not detectably along the sugarcane *Cenpc1 *lineage (K_a_/K_s _= 0.23). PAML analysis also confirmed that the carboxy-terminal region of maize *CenpcA *is adaptively evolving (χ^2 ^= 7.8; *p *= 0.02 for the data-driven model over the null model) with K_a_/K_s _= 1.4. Thus, different methods of analysis demonstrate that CENP-Cs are adaptively evolving in an episodic fashion in grasses that have multiple CENP-C copies.

Maize *CenpcA *is co-expressed with another *Cenpc *gene, *CenpcB *[25], for which incomplete sequence information is available (AF129858, AW062057, and AY109432). The available *CenpcB *sequence begins in exon 5 and continues through exon 14, and has seven in-frame indels relative to *CenpcA*. We found negative selection in a region from the end of exon 5 through the first half of exon 6 (*CenpcA *codons 205-358, *p *< 0.01), and positive selection from the end of exon 6 through exon 7 (codons 403-492, *p *< 0.01). Elsewhere the null hypothesis could not be rejected. Comparing *CenpcB *with *S. bicolor*, K_a _= 0.13 and K_s _= 0.11, and there are six in-frame indels between the sequences. We found positive selection in the first half of exon 6 (*Sorghum *codons 273-327, *p *< 0.03) and negative selection from exon 10 through the first few codons of exon 13 (codons 537-619, *p *< 0.02). Elsewhere the null hypothesis could not be rejected. In summary, regions under negative selection in other grass *Cenpc*s can be under positive selection in *CenpcB*, and regions under positive selection in other grass *Cenpc*s are under negative selection or evolving neutrally in *CenpcB *(Figure 3b), suggesting that CENP-CB has been subjected to different selective forces since its divergence from CENP-CA.

Just as gene duplication can result in different selective pressures on the two genes, duplications within a gene can lead to specialization and thus can change selective pressures on the region. Such specialization appears to have occurred between the anciently duplicated region encoded by exons 9-10 and 11-12 (Figure 6a). In maize, sugarcane, and *Sorghum *we detected negative selection for exons 9-12, but in the more recent duplication of exons 9 and 10 in wheat and barley we detected positive selection in a region from the last codon of the first copy of exon 10 to the first four codons of exon 12 (*p *< 0.01). Additional windows in exons 9p-10p and 12 had K_a _*>*K_s _(*p *> 0.05), suggesting that most of the duplicated region has been evolving adaptively (Figure 2d). In contrast, in the adjacent conserved carboxy-terminal region (corresponding to *CenpcA *codons 625-690), we detected only negative selection (*p *= 0.01), as though exon duplication allowed for adaptation.

We find an approximate correspondence between adaptively evolving regions in angiosperms and those in mammals that overlap DNA-binding and centromere-targeting regions. Although no DNA-binding regions have been experimentally determined for plant CENP-Cs, the correspondence with animal CENP-Cs suggests that the different regions play comparable roles. One of the corresponding adaptive regions is repeatedly duplicated in grasses, and the distribution of basic residues in exons 10 and 12 suggests that the repeat unit binds DNA. A parallel situation again appears to be found for the amino-terminal tails of some *Drosophila *CenH3s, which contain repeats of a minor groove-binding motif that are thought to provide DNA compositional preference [12]. Thus, both plant and animal CENP-Cs show adaptively evolving features that parallel those found in CenH3s.

### Yeast *MIF2 *is under negative selection

If positive selection for CENP-Cs in plants and animals is related to centromere complexity, then we would expect conventional negative selection to operate in organisms such as budding yeast, which have simple centromeres. The *MIF2 *genes of *Saccharomyces cerevisia*e [26] and *S. paradoxu*s [55] are 93% identical in amino-acid sequence, with K_a _= 0.036 and K_s _= 0.38. In sharp contrast to all pairwise comparisons of plant and animal *Cenpc *genes, K_a _was much less than K_s _for all of the 99 bp windows of yeast *MIF2*, indicating that it is under negative selection throughout its length (*p *< 0.001). In all pairwise comparisons among these two species and the additional species *S. mikatae *and *S. bayanus *[55], we consistently found evidence of negative selection with K_a _<< K_s _(range of K_a_, 0.036-0.093; range of K_s_, 0.38-0.82). We also found strong negative selection for all 99 bp windows in pairwise comparisons of yeast CenH3 (Cse4p; data not shown). Thus, adaptive evolution of both CenH3s and CENP-Cs appears to be limited to organisms with complex centromeres.

### Meiotic drive model of centromere evolution

We have demonstrated that CENP-C has been adaptively evolving in multiple lineages of both plants and animals, a feature that had been previously shown for some CenH3s. Thus, the occurrence of adaptive evolution appears to be a general feature of proteins that bind to complex centromeres. Recurrent adaptive evolution implies an arms race, and an arms race involving centromeric DNA-binding proteins is remarkable given that centromeres have a conserved function. But centromeric DNA is rapidly evolving in plants and animals, so adaptation of the major centromere DNA-binding proteins would maintain an interface with the conserved kinetochore machinery. Indeed, regions of CENP-C that show evidence of positive selection include DNA-binding and specificity regions, in parallel with previous findings for *Drosophila *and *Arabidopsis *CenH3 [9,11,44].

A 'meiotic drive' model has been proposed to explain the rapid evolution of centromeric DNAs and CenH3s [1]. According to this model, centromeres compete during female meiosis for inclusion in the single meiotic product that becomes the egg nucleus and so gets transmitted to the next generation. In both animals and seed plants, which of the four meiotic products becomes the egg nucleus is determined by its position in the female tetrad. A centromere variant will increase in the population if it achieves an orientation in female meiosis resulting in its inclusion in the egg nucleus more frequently than its competitors. For example, an expansion of a satellite array may lead to a 'stronger' centromere variant with an expanded kinetochore that attracts more microtubules and results in a slightly greater probability of a favorable orientation in female meiosis. The mechanism of such orientation is unknown, but in some insects and plants the female meiotic spindle has an asymmetric distribution of microtubules or is monopolar [56], so a stronger centromere variant might better capture the favored pole. The new variant will therefore increase in the population and eventually become fixed. This meiotic drive process ('centromere drive') can account for the rapid evolution and complex structure of centromeric DNA. As a rare new variant spreads in the population, however, disparities in centromere strength may interfere with fertility in males, where the four meiotic products contribute equally to the next generation. Mutations in CenH3 that restore centromere parity in meiosis will therefore be selected in males, resulting in the adaptive evolution of CenH3 and suppression of the meiotic drive of centromeric DNA. Recurrent cycles of meiotic drive by centromere variants, or centromere drive, and suppression by CenH3 mutations would result in the observed rapid evolution of both centromeres and CenH3s.

The lack of evidence for adaptive evolution in CenH3s from mammals and grasses does not seem to fit this scenario. But the extensive positive selection on the corresponding CENP-Cs provides a ready explanation for the absence of an adaptive signal for CenH3. The meiotic drive model predicts that over evolutionary time any mutation that restores centromere parity will be selected, suggesting that proteins besides CenH3 - and in particular other kinetochore proteins that contact centromeric DNA - may be positively selected to suppress centromere drive. Our demonstration of the adaptive evolution of CENP-C, especially in DNA-binding regions, fulfills this prediction of the centromere drive model. Apparently, in mammals and grasses CENP-C performs the function of a suppressor of meiotic drive.

The large size and lack of sequence conservation of CENP-Cs make them much larger mutational targets for suppression than CenH3s. Moreover, PAML analysis suggests that a larger proportion of CENP-C than CenH3 residues are evolving adaptively. Mammalian CENP-A consists of a well-conserved histone-fold domain with only a short unconstrained tail region. Conversely, *Drosophila *species have the longest CenH3 tails known [12] but lack any identifiable CENP-C homologs. It is tempting to speculate that the interaction of the long CenH3 tail of *Drosophila *with centromeric satellites compensates for the absence of CENP-C and permitted its loss. This might explain why *Drosophila *CenH3s localize in a species-specific manner [44], whereas human CENP-A can be functionally replaced by its budding yeast CenH3 counterpart [57].

Centromere drive may have important consequences for karyotypic evolution. Centromeres of two acrocentric chromosomes frequently fuse (Robertsonian translocations), and metacentrics often misdivide to yield two acrocentrics. In humans, there is a bias in favor of Robertsonian translocations over their homologous acrocentric pair when transmitted by females, and male carriers have reduced fertility [58]. This general sterility of Robertsonian males is consistent with centromere drive underlying post-zygotic reproductive isolation in emerging species [1]. Centromere drive provides a mechanism for the tendency of karyotypes to be either mostly metacentric or mostly acrocentric [59] and for the karyotype-specific accumulation of selfish B chromosomes in mammals [60]. Our finding that CENP-Cs, like CenH3s, evolve adaptively addresses a perceived shortcoming of the centromere drive model for post-zygotic reproductive isolation: mutations that rescued hybrid sterility did not map to the *Drosophila *CenH3 gene [61,62]. The fact that CenH3 is not the only adaptively evolving centromere protein indicates that there are multiple candidate drive suppressors that might rescue hybrid sterility when in a mutant form.

In contrast to CENP-Cs of plants and animals, yeast Mif2p appears to have evolved entirely under negative selection. This is consistent with Mif2p interacting with a stable centromere, rather than one that is rapidly evolving. In accordance with this observation, budding yeast centromeres are determined by the presence of a consensus DNA sequence that includes binding sites for the Cbf1 and CBF3 proteins [49]. The consensus DNA sequences and their binding proteins are recognizably similar in yeasts as distantly related as *Candida glabrata *and *Kluyveromyces lactis*, which have greater average divergence from budding yeast in protein sequences than mammals have from fish [63]. We attribute this extreme conservation of centromere sequence to optimization of the DNA-protein interactions at the centromere. Such optimization would be inevitable in fungi that produce equivalent gametes in a tetrad. No such optimization would occur when centromeres compete at female meiosis I for a favored orientation. Seed plants and animals evolved female meiosis independently, so the parallels that we see for evolution of CenH3 and CENP-C would reflect parallel evolutionary forces in these two ancient lineages.

## Materials and methods

### DNA clones and sequencing

Genomic DNAs and cDNAs were obtained from several sources (Additional data file 2). The *A. thaliana *cDNA was amplified from a cDNA pool from whole plants of the ecotype Columbia, and the *A. arenosa *cDNA was amplified from a cDNA pool from leaves of Care-1 [64]. Both of these cDNAs were amplified using the same primers: 5'-GGAATTTTCCGGTGATTTAGATG-3', which terminates in the initiation codon, and 5'-TGATCACAAGAGGATGGTTGA-3', from the 3' untranslated region of the *A. thaliana *genomic *Cenpc *sequence. Genomic DNAs from wheat and barley were generously provided by Andreas Houben. Exons 9p-10p and the intervening intron 9p were amplified from both wheat and barley using the primers 5'-AGATGAACCAATCCATCCAC-3' and 5'-AAATTCGTTTTCCTCTCTTTGCT-3'. Likewise, 9q-10q and intron 9q were amplified with the primers 5'-AGATAAGCCAATCCATACATCA-3' and 5'-CCCCTCTTTTCATTCTCTTCAA-3'. The first and last of these four primers were also used to amplify both exon pairs as a unit to confirm their contiguity and to determine the genomic sequence around the junctions of intron 10p with exons 10p and 9q. Amplifications used High Fidelity Platinum *Taq *polymerase (Invitrogen, Carlsbad, USA). The amplified fragments were cloned using the pCR2.1-TOPO-TA cloning kit (Invitrogen) according to the manufacturer's instructions. Sequencing was carried out using ABI Big Dye sequencing on both strands of all reported sequences. Sequencing primers were standard vector primers or were designed using Primer 3 [65]. Sequences were assembled using Sequencher 4.1.2 software [66]. Accession numbers of sequences are given in Additional data file 2.

### Sequence analyses

Sequence similarities of genes and their encoded proteins were identified using the NCBI BLAST server [35,67], as well as by use of Gramene [68] and the TIGR Gene Indices [69]. Translations and sequence manipulations utilized the Sequence Manipulation Suite [70,71]. Alignments of coding and amino-acid sequences were performed using the European Bioinformatics Institute Clustal W Server [72,73], with adjustments by hand to take account of splice-site alignment. Conservation in alignments was displayed using MacBoxShade 2.1 (MD Baron, Institute for Animal Health, Surrey, UK). Protein blocks were made, displayed, and compared using the Multiple Alignment Processor, sequence Logos, and LAMA [74] programs on the Blocks WWW Server [75]. To make blocks from grass exons 9-12, gaps in ClustalW alignments were first filled with Xs, which do not appear in subsequent sequence Logos representations. Gene models of exon-intron boundaries were made by alignment of cDNAs with identical or homologous genomic sequences, as well as by splice-site prediction using the NetGene2 server [76,77].

K-estimator [31] was used to estimate K_a _and K_s _in comparisons of *Cenpa/CENH3 *or *Cenpc *genes from pairs of closely related species. Prior to analysis, gaps were removed from the coding sequences as indicated by the amino-acid alignments.

We estimated K_a _and K_s _for windows of 99 nucleotides, positioned every 33 nucleotides. For candidate regions of positive selection, we determined the confidence intervals of K_a _and K_s _under the null hypothesis that K_a _is equal to K_s _using the default parameters (1,000 replicates). For individual windows of 99 nucleotides, or for regions defined by contiguous groups of overlapping windows, limited trial and error suggested that statistically significant positive selection was not supported if K_a_/K_s _< 1.5. Therefore, to find evidence of positive selection, we determined the confidence intervals for regions defined by sets of overlapping or immediately adjacent 99 nucleotide windows with K_a_/K_s_ ≥ 1.5. For regions with K_s _= 0, one or more flanking windows with K_s _> 0 were included in the region analyzed, regardless of the value of K_a_, so that a value for K_a_/K_s _could be defined. Similarly, we looked for statistically significant negative selection for regions defined by overlapping or adjacent 99 nucleotide windows with K_a_/K_s _≤ 0.67. The codeml program of PAML version 3.13d [40] was also used to test for positive selection and to estimate K_a_/K_s _ratios as previously described [11].

## Additional data files

The following are provided as additional data files. Additional data file 1, containing Table S1, reports accession numbers for selected *Cenpc *ESTs and genomic sequences from GenBank. Additional data file 2, containing Table S2, reports accession numbers for *Cenpc *cDNAs and amplified genomic sequences. Additional data file 3, containing Figure S1, displays the conservation of the exon containing the CENPC motif.

## Supplementary Material

Additional data file 1 Table S1 reports accession numbers for selected *Cenpc *ESTs and genomic sequences from GenBank Click here for additional data file

Additional data file 2 Table S2 reports accession numbers for *Cenpc *cDNAs and amplified genomic sequences Click here for additional data file

Additional data file 3 Figure S1 displays the conservation of the exon containing the CENPC motif Click here for additional data file

## Figures and Tables

**Table 1:** Pairwise comparison of mouse and rat *Cenpc *genes

Human	Mouse	Rat	Selection
1-86	1-84	1-77	+*
109-248	107-218	100-236	+**
239-304	208-273	226-291	-**
294-353	263-321	281-335	+*
411-455	377-420	391-434	+**
445-497	410-464	424-478	- (0.06)
487-552	454-519	468-533	+**
565-670	531-633	545-643	+**
671-790	634-754	644-764	-**
858-934	821-897	831-907	-*

+ denotes K_a _> K_s_; -, K_a _< K_s_; * *p *< 0.05; ** *p *< 0.01. Number ranges represent codon positions based on the complete coding sequences prior to removal of indels for alignment. Human codon positions are given for comparison with previous functional studies. Number in parentheses is a *p *value greater than 0.05.

**Table 2:** Regions of selection in pairwise comparisons of maize *CenpcA, Sorghum bicolor Cenpc*, and sugarcane *Cenpc1*

Exons	Direction of selection	Maize *vs. Sorghum*	Maize *vs. *sugarcane	*Sorghum vs. *sugarcane
1	+	12-44	12-44	1-42
		+	+ (0.17)	+ (0.04)
1-5	-	34-165	23-176	87-163
		-	-	-
4-6	+	155-253	166-253	153-317
		+	+	+
6	*		232-286	
			-	
6	-	298-363	298-363	298-352
		-	-	- (0.13)
6	+	353-409	342-407	397-431
		+	+	+ (0.06)
6-12	-	410-621	397-630	432-579
		-	-	-
12-14	*	611-687	609-685	591-700
		+	+	-

+, K_a _> K_s_; -, K_a _< K_s_; *p *≤ 0.01 except where given in parentheses. * Direction of selection varies with lineage. Regions of selection are identified by codon positions based on the sequence of maize *CenpcA*.
