# Supplementary material for: Adaptive evolution of centromere proteins in plants and animals
Source: J Biol. 2004 Aug 31;3(4):18. doi: 10.1186/jbiol11 (PMC549713; doi:10.1186/jbiol11)
Supplement: Additional data file 1 — Table S1 reports accession numbers for selected Cenpc ESTs and genomic sequences from GenBank [file jbiol11-s1.pdf]

Additional data file 1

Table S1

Selected *Cenpc* ESTs and genomic sequences from GenBank

Organism	ESTs	Genomic sequences (locus)
Rat	CB327827, BQ190551, BG669698	AC095997
Human		AC104806, AC109356, AF151723
Chimpanzee		(From trace archives)†
Dog	BU748146, BU748145	
Cow	CB428746	
Schizosaccharomyces pombe		AL023288, AL109834 (SPBC1861.01c)
Cryphonectria parasitica	CB690425	
Physcomitrella patens	BJ167802*, BJ159529*	
Poplar	BI135594, BI136606, etc.	
Beet	BQ488389*	
Arabidopsis thaliana	AV559306	AC013453 (At1g15660)
Apple	CN489643	
Barrel medic	BE325476	AC138580*
Soybean	AW780955	
Scarlet runner	CA902018	
Lotus	AW720394	
Zinnia	AU290185	
Sunflower	BQ916764*	
Lettuce	BQ996789, BQ999967*	
Potato	BF054220, BQ115396, BE343178, BQ509271	
Tomato	AI485238, AI771307, AI484910, AW622731, BI925195	
Maize	AW062057, AY109432, etc.	BH215393, BZ803155, CC445929, CC605630, CG179177, CG008398*, etc.
Sorghum bicolor	BG355465*, BG355791*, CF072297, CD228196*, CD228224*	CL157864, CL193099, CL193100
Sorghum propinquum	BF421310	
Sugarcane	CA256451, CA169588, CA083292, CA122448, CA065654, CA294311, CA079147, CA148005, CA191044, CA138332. CA098410, CA294240, CA256540, CA065728, CA122508, CA16966, BQ533265	
Rice	CB000111, C27347	AP002897 (P0686E09.20)
Barley	BU992050, BU998115*, CA026000 BM098142, AJ474427	
Wheat	BQ280552, AL820601*, CA658451, CA741440, BJ303473, BJ309345, BJ302979, BJ308833, BJ302988, BJ308841, BJ277765, BJ282957	

*Probable pseudogene or improperly spliced; †the chimpanzee *Cenpc* gene structure was predicted by alignment with the human, mouse, and rat genes, including alignment of intron positions [29]. Chimpanzee *Cenpc* may differ at the beginning of exon 5, where a G/C transversion may necessitate the use of a splice acceptor sequence (a**c**tgccag^AAA) that is two codons downstream from the site used in human *Cenpc* (a**g**^TGC CAG AAA).
