# Supplementary material for: Adaptive evolution of centromere proteins in plants and animals
Source: J Biol. 2004 Aug 31;3(4):18. doi: 10.1186/jbiol11 (PMC549713; doi:10.1186/jbiol11)
Supplement: Additional data file 2 — Table S2 reports accession numbers for Cenpc cDNAs and amplified genomic sequences [file jbiol11-s2.pdf]

Additional data file 2

Table S2

***Cenpc* cDNAs and amplified genomic sequences**

Organism	cDNAs or Genomic Amplicon	Accession	Source
Rat	UI-R-FS0-crt-p-12-0-UI	AY693786	[78]
	UI-R-DQ1-ckv-e-04-0-UI	AY693787	[78]
	**DRNAAIB08**	AY693788	[79]
Cow	FQY8021 Row:G Col:8	AY693782	[80]
A. thaliana	**CenpcCol1**	AY693798	[64]
A. arenosa	**CenpcCare1**	AY693778	[64]
Barrel medic	**NF089E04ST**	AY693784	[81]
Soybean	Gm-c1037-952	AY693794	[82]
Beet	**L-11-3***	AY693781	Bernd Weisshaar
Potato	cSTB39G13*	AY693785	[83]
Tomato	cTOB4K16	AY693799	[84]
Sugarcane	**SCBFAD1047C06 (*Cenpc1*)**	AY693795	[85]
	**SCEQRT2025C08 (*Cenpc2*)**	AY693796	[85]
	**SCEZRZ1015G07 (*Cenpc1*)**	AY693797	[85]
Sorghum bicolor	**EM1_16_G07_A002***	AY693791	[86]
	**FE1_7_B02 _A002**	AY693792	[86]
	CCC1_6_F09_A007*	AY693790	[86]
Sorghum propinquum	**FM1_7_B04 _A003**	AY693793	[86]
Rice	S345W-D05	AY693789	Benildo G. de los Reyes
	C51663_1A	AY693783	[87]
Barley	exons 9p-10p	AY693780	Andreas Houben
	exons 9q-10q	AY693779	Andreas Houben
Wheat	WHE3002_C07_E14	AY693800	[88]
	C05_O232_plate_15*	AY693801	[89]
	exons 9p-10p	AY693802	Andreas Houben
	exons 9q-10q	AY693803	Andreas Houben

*Probable pseudogene or improperly spliced; **bold**, full-length cDNA.
