# Supplementary material for: Adaptive evolution of centromere proteins in plants and animals
Source: J Biol. 2004 Aug 31;3(4):18. doi: 10.1186/jbiol11 (PMC549713; doi:10.1186/jbiol11)
Supplement: Additional data file 3 — Figure S1 displays the conservation of the exon containing the CENPC motif [file jbiol11-s3.pdf]

Additional figure

(a)

```
Exon boundaries       |                                    |

Mouse            LHHKL|VLPSN-SPNVRRSNRIRLKPLEYWRGERVDYQESSS|GQLVL
Chicken          GRQKI|VLPSN—TPNVRRTKRIRLKPLEYWRGERVTYTLKPS|GRLLI

Arabidopsis      RKSLA|AAGTKIEGGVRRSTRIKSRPLEYWRGERFLYGRIHE|SLTTV
Rice             RKSLA|DAGLTWQAGVRRSTRIRSKPLQHWLGERFIYGRIHG|TMATV
                                ________________________
                                      CENPC motif
```

(b)

```
Mouse               ag|TA CTG CCC.................TCG TCG G|gt
Chicken             ag|TG CTG CCT.................CCT TCA G|gt

Arabidopsis         ag|CT GCT GGT.................CAT GAG A|gt
Rice                ag|AT GCG GGT.................CAT GGA A|gt
```

Figure S1
Conservation of the exon containing the CENPC motif. **(a)** Alignment of amino-acid sequences encoded by exons containing the CENPC motif in mouse, chicken, *Arabidopsis,* and rice. **(b)** The flanking splice junctions are positioned in the +1 reading frame.
